# Assessment of Overheating Risk in Gynaecology Scanning Rooms during Near-Heatwave Conditions: A Case Study of the Royal Berkshire Hospital in the UK

**DOI:** 10.3390/ijerph16183347

**Published:** 2019-09-11

**Authors:** Hannah Gough, Samuel Faulknall-Mills, Marco-Felipe King, Zhiwen Luo

**Affiliations:** 1 Department of Meteorology, University of Reading, Reading RG66UR, UK; 2 Royal Berkshire Hospital, Reading RG1 5AN, UK; Samuel.Faulknall-Mills@royalberkshire.nhs.uk; 3 School of Civil Engineering, University of Leeds, Leeds LS6 1AN, UK; M.F.King@leeds.ac.uk; 4 School of the Built Environment, University of Reading, Reading RG66UR, UK; z.luo@reading.ac.uk

**Keywords:** hospital, overheating, temperature, heatwave, climate, case study

## Abstract

Hospital buildings in the UK are at particular risk to rising summer temperatures associated with climate change. Balancing the thermal needs of patients, staff, and visitors is a challenging, complex endeavour. A case study of the ultrasound area of the Royal Berkshire Hospital’s Maternity and Gynaecology building is presented, where temperatures were measured for 35 days in waiting areas, staff offices, and ultrasound scanning rooms, aiming to assess the overheating risk posed to occupants. Local external temperature measurements were used for comparison whereby determining the indoor-outdoor environmental connection. Results show that most rooms had already breached standard overheating thresholds within the study period. Anthropogenic and waste heat from equipment has a noticeable effect on indoor temperatures. Local air-conditioning helped reduce the peaks in temperature seen between 14:00 and 17:00 for similar scanning rooms but is in contradiction to the National Health Service’s sustainability plans. Several low-level solutions such as improved signage, access to water, and the allocation of vulnerable patients to morning clinics are suggested. Barriers to solutions are also discussed and the requirement of sufficient maintenance plans for cooling equipment is empathised. These solutions are likely to be applicable to other hospital buildings experiencing similar conditions.

## 1. Introduction

The effect of climate change on indoor summertime temperatures within UK buildings is likely to lead to overheating, due to predictions of the season being warmer and drier [1]. Effects of indoor conditions in buildings upon humans are multiple, interlinked, and complex [2]. The effect of thermal stressors on human performance is complex and dependent on the duration of the exposure, task type, and intensity of the stressor [3,4,5]. Data from the summer of 2006 highlights that external temperatures over 25 °C could lead to increased mortality especially for those most at risk (elderly and young children) [1]. Short term exposure to high temperatures during heatwaves may have a detrimental effect on birth weight and birth length [2].

While prolonged exposure to extreme temperature affects cognitive ability, decrements to cognitive performance and effects on illness absence can also occur with far milder fluctuations of temperatures [6,7,8]. Peak productivity was found to occur between 21 and 22 °C (dry bulb, relative humidity not defined), where the performance began to decrease above 24 °C [2]. Warm discomfort affects performance more rapidly than cool discomfort [9]. Weather-related high ambient temperatures are associated with an increased risk of work-related injury, especially in the physically active, with heat being a well-described occupational hazard [10,11,12,13,14,15,16]. Work by [17] highlights that even in more extreme temperatures, the general public is not aware of the risks of overheating causes.

The health-care sector is of particular concern due to hospitals being occupied by vulnerable patients and being a refuge to those most affected by the heat during extreme heat events [18]. The Adaptation Sub-committee of the UK Committee on Climate Change has identified the risk of overheating and poor thermal comfort in hospital buildings [19]. Hospitals must provide respite from the summer heat for the most vulnerable people at precisely the times of the year when it is most difficult to do so and when demand may surge [18,20]. The National Health Service (NHS) faces a two-pronged challenge: delivering safe and resilient environments within a changing climate whilst meeting ambitious carbon reduction targets, meaning that air-conditioning, and/or mechanical ventilation systems are not the medium to long-term solution [21,22,23,24]. The building stock of the NHS is mixed, with some stock being mechanically ventilated and others relying on only natural ventilation which although cheaper, may be less effective and unsuitable for certain areas [25].

Increased patient footfall and resultant staffing workloads compared to initial design expectations mean that hospitals face additional challenges, including the wide range of different activities that occur within them, a workforce that functions 24 h a day, the storage of medications, and the increased amount of heat-sensitive and heat-producing equipment [26]. Creating a comfortable thermal environment within a hospital is complex often due to the opposing needs activities and clothing levels of individuals, where clinical staff, administration staff, visitor and patient comfort must be balanced. Staff are more likely to occupy the same environment, whilst visitors will be more transient, though both have normal thermal comfort needs when compared to the patients, who may be sensitive to extremes and sudden temperature changes due to pregnancy, age, or sickness [25]. Heat-stroke in staff must also be considered, especially if personal protective equipment has to be worn and workloads have increased due to increased demand during a heatwave [27,28].

Current research tends to focus towards single hospital case studies due to difficulties working within such an environment [22,29,30,31,32]. The study of environmental conditions within a hospital is often focused on infection control across the entire site (e.g. [22,23,24]), or overheating (e.g. [23,24,25]), often with a focus on the ward environment, where inpatients spend most of their time. However, research or case studies in other areas of the hospital are rarer (e.g. [28]) though they will also have an effect on patient treatment, comfort and satisfaction.

This paper aims to analyse the temperatures experienced by patients within ultrasound scanning rooms in the Royal Berkshire Hospital’s (RBH) Maternity and Gynaecology (M and G) building in Reading, UK, under near-heatwave conditions and explores a broad range of potential solutions, termed low-hanging fruit. This paper also highlights the complex disconnect between indoor and outdoor environments, complicated by the inclusion of neighbouring buildings, meaning that local measurements are often required in order to understand local flows around the building [33,34]. These overheating problems are not unique to the RBH Maternity and Gynaecology building as shown by [35,36] and the suggested solutions may benefit other NHS Trusts.

Heat mitigation strategies are limited by the NHS’s focus on bed availability and infection control: the need for strict infection control may limit the installation of ventilation systems. There is no uniform solution to overheating due to the sheer variety and number of healthcare buildings, each with unique building management plans and needs [18]. More than a fifth of the NHS building portfolio was built prior to the birth of NHS in 1948 and a few buildings date back to 1700 [37].

Other considerations are tight budgets, so solutions must be easy to apply, cheap to install and cheap to maintain, with low or no energy costs: creative, simple, and non-intrusive solutions [25]. The variety of buildings and needs of the NHS suggest that specific measurements within areas of concern can help identify and put boundaries on the problem, allowing for solutions to be generated. This study provides a case study of an ultrasound scanning area, identified by staff as frequently overheating and general solutions which could be applied across a wide variety of NHS building stock.

## 2. Materials and Methods

**Figure 1:**
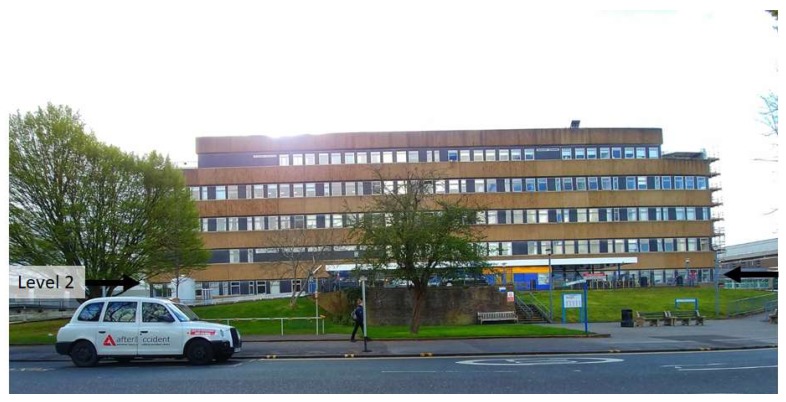
Photo of the Maternity and Gynaecology building taken from across the road. The black panels seen around the windows are asbestos. Level two is the ground floor of the building, highlighted by the black arrows, with the two entrances (Figure 2) located under the white awning.

Investigations at the Royal Berkshire Hospital’s (RBH) Maternity and Gynaecology (M&G) building, Reading, UK, (Figure 1) brought to light that staff and patients were falling ill and in one case fainting due to high indoor temperatures, which occurred outside of heatwave conditions (>31 °C daytime temperature and 16 °C max night time temperature [18]). Upon identifying this risk, some ad-hoc, local air-conditioning devices had been implemented, allowing for comparisons between rooms. This research was undertaken as a response to staff raising concerns about patient safety and high indoor temperatures. Lomas et al. [21] describe the variation in what is deemed overheating within indoor environments, noting that literature was broadly consistent in placing an upper threshold of 27–28 °C and permitting a small number (or percentage, normally 1%) of occupied hours to exceed this. For example CIBSE (Chartered Institute of Building Services Engineers) defines overheating as temperatures exceeding 25 °C for >5% and/or 28 °C for >1% of occupied (working) hours [38].

The department was given conflicting advice about the use of disinfectant units (Throphon): they may not be operated in temperatures >27 °C due to manufacturer limits, however, it has not been made clear the risks of operation above this limit or the effect on the efficiency of disinfectant. Another form of probe disinfectant (Tristel wipes), has had to be discontinued from use due to fumes, causing staff to become ill, with a tightness in the chest and irritation in the throat being symptoms described by the manufacturer when fumes are inhaled [39]. This is likely due to the wipes being heated, either by waste heat from the machines, or being used to disinfect warm surfaces [39]. The manufacturer states that wipes should be stored and used in a cool, well-ventilated area, with heat listed as a condition to avoid, though no specific temperatures are given [39]. Anecdotal evidence suggests that wipes were being stored on top of warm equipment for ease.

This building was built in the 1960s and constructed of mostly concrete and asbestos with no façade shading. Windows are single-glazed with ill-fitting frames. The building was not designed to facilitate the anthropogenic, IT, and electronic heat loads seen in a modern M and G department. There is a mechanical ventilation system mainly used for heating, but it is turned off in the Summer months for cost-saving unless heatwave conditions are declared, or extreme temperatures are reported to estates, though there is a delay between reporting and activation.

**Figure 2:**
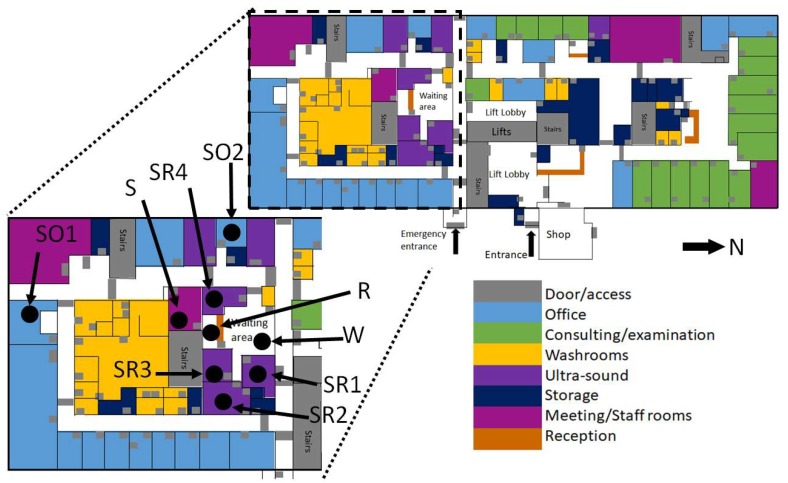
Floor plan of level 2 of the Maternity and Gynaecology building, colour coded by room use. Total area: 2162.52 m^2^. Cutaway shows the focus area of the study and the symbols for sensors and locations are described in Table 1. The map is based on building plans provided by RBH.

The focus area of the study was the waiting/reception area (capacity: 20–30 people) and staff break room (capacity: 10 people) associated with the ultrasound scanning rooms (6 rooms, 4 sealed, 2 naturally ventilated) on Level 2, the ground floor (Figure 1 and Figure 2). The only rooms with windows are the ones directly connected to the building façade, in this case, the two staff offices (Figure 2, marked as SO1, SO2 respectively). Only the scanning rooms without direct natural ventilation (four) are monitored. These are positioned within the centre of the building (Figure 2). Room 3 (Marked as Sensor SR3) has air-conditioning available to use, with a portable air-conditioning unit also being deployed (unknown date of deployment and operation times) in the waiting room in front of the reception desk (Figure 2). Sensor naming convention is shown in Figure 2. Occupants of these areas include admin, cleaning, and clinical staff (all day), patients and pregnant patients (<5 h stay), all of whom will be negatively affected by high indoor temperatures [40]. Varying environmental conditions have also been reported across the entire floor and building (Figure 2).

**Figure 3:**
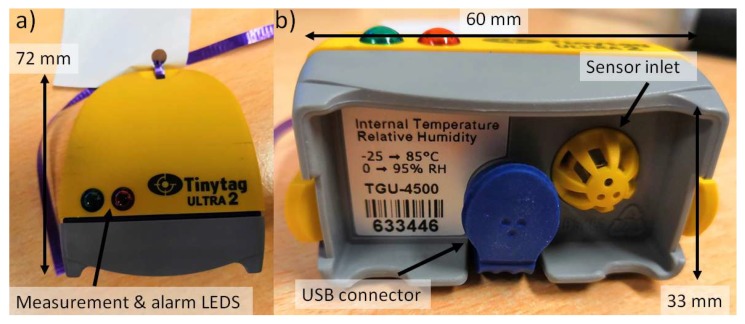
Images of the Tinytag sensors deployed (**a**) plan view and (**b**) closeup of the sensor base where the inlet is visible. All sensors had a piece of paper attached describing what they were measuring and who to contact if issues arose.

Temperature and humidity within the internal environment were measured using nine Gemini Tinytag Ultra 2 TGU-4017 temperature loggers (temperature range −40 to 85 °C) chosen for their splash-proof case, self-contained design and USB (Universal Serial Bus) connection (Figure 3). Where possible sensors were positioned out of direct sunlight (only a problem for the Staff Offices, see Figure 2) and away from windows or any office equipment which may generate waste heat. Within the waiting room, the sensor was placed in the corner of the room, out of sight, but within the area where patients sit and wait.

The Tinytag sensors were none-intrusive and battery-powered to reduce trip hazards. Temperature logging commenced on the 26/07/2018 at 13:00 with all sensors being programmed to measure temperature and humidity every five minutes (accuracy: 0.1 °C, 5%). Three hours of calibration were undertaken beforehand. Measurements in-situ were stopped on 30/08/2018 at 13:00, with a calibration being undertaken between 13:00 and 17:00. Calibration on the instruments involved placing all the instruments into a controlled temperature space and intercomparing results. All data included here has undergone correction.

**Figure 4:**
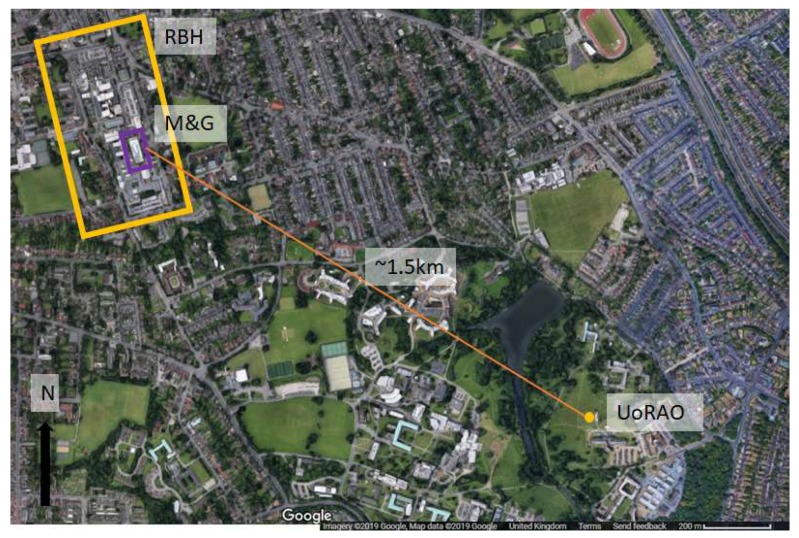
Location of the University of Reading Atmospheric Observatory (UoRAO, dot) in comparison to the Royal Berkshire Hospital site (RBH, box). The Maternity and Gynaecology (M and G) building (Figure 1) is highlighted within the RBH site.

**Figure 5:**
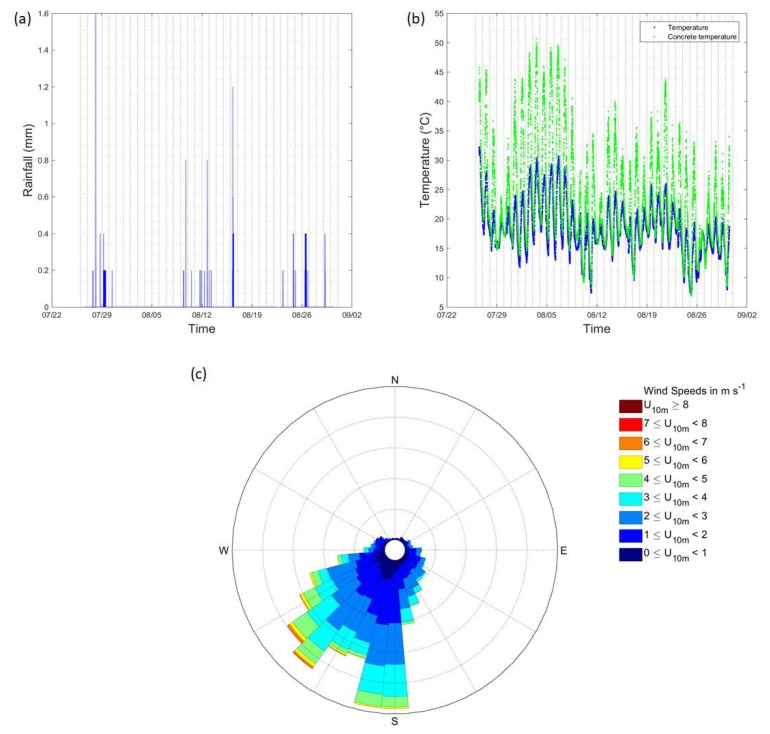
Observations from UoRAO for the study period. (**a**) rainfall, (**b**) temperature and concrete temperature, and (**c**) wind direction and 5-min wind speed at 10 m (meteorological standard). Vertical grey lines denote midnight of each day.

Reference external temperatures were measured at the University of Reading’s Atmospheric Observatory (UoRAO), also at five-minute intervals as part of the automated system (accuracy: 0.1 °C). The temperature of concrete was also logged. The climate conditions of Reading can be classed as temperate. UoRAO is approximately 1.5 km as the crow flies from the Maternity and Gynaecology building (Figure 4). For details on the experimental set up of the UoRAO and measurements available see [41]. Whilst this distance is small, the external temperatures around the hospital site may be slightly warmer due to the urban heat island effect. Figure 5 details the background weather conditions throughout the campaign, with little rain being recorded (Figure 5a). Low wind speeds (95 % were <6 m s^–1^) were recorded throughout the study period and for most of the study period, the wind direction remained within the prevailing sector for the area (Figure 5c).

Indoor temperature data is available here: http://dx.doi.org/10.17864/1947.224. Measurements from the atmospheric observatory can be obtained on request from the Department of Meteorology, University of Reading. See https://research.reading.ac.uk/meteorology/atmospheric-observatory/ for details.

## 3. Results

**Figure 6:**
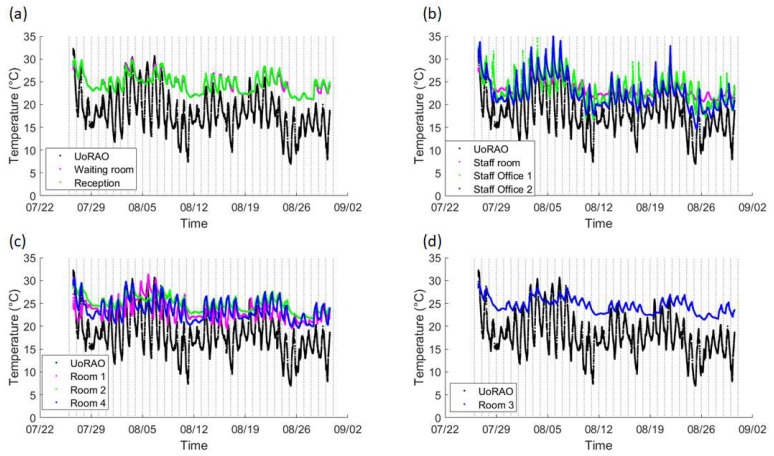
Overview of the temperatures recorded for the entire study period. Vertical lines denote midnight of each day. For clarity, similar rooms are plotted on one subplot, (**a**) Observatory (UoRAO), waiting room and reception, (**b**) UoRAO and Staff room and offices, (**c**) UoRAO and the naturally ventilated scanning rooms (Room 1,2,4) and (**d**) UoRAO and room 3, the air-conditioned room. All plots have the same axis.

**Figure 7:**
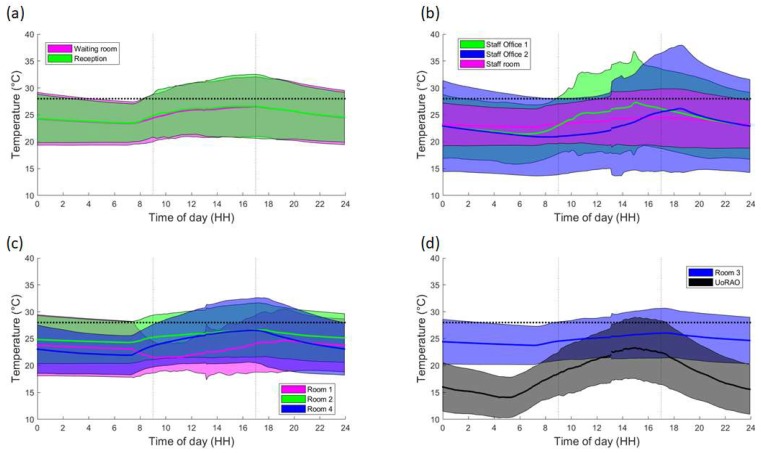
Time-averaged trend of weekday temperatures within the rooms. Line denotes the mean temperature per five minutes, with the shading denoting three standard deviations. (**a**) Waiting room and Reception, (**b**) Staff offices and the Staff room, (**c**) Rooms 1, 2 and 4 and (**d**) Room 3. Note that for clarity the UoRAO data is on plot d). Clinic hours are highlighted by the dashed vertical lines. The CIBSE threshold of 28 °C is shown in the thick horizontal dashed line.

A diurnal cycle is present for all rooms, with more extreme temperatures being seen in the Staff offices, due to them being located on the building’s exterior (Figure 2, Figure 6b). The staff offices are also where the lowest indoor temperatures are recorded during the evenings, due to the rapid release of heat from the building’s surface and potential ventilation. The waiting room and reception area (Figure 6a) both record similar conditions due to them being linked, with the waiting room having lower minimum temperatures due to it being closer to the main entrance (automated sliding double doors ~1.5 m across) and the effect of the temporary air-conditioning unit (Figure 2). The staff room also displays a similar trend, though with larger daytime spikes due to the lack of ventilation because of closed doors for privacy and security reasons (Figure 7b). The ultrasound scanning rooms all show slightly different behaviours, with room three displaying lesser peaks due to the use of air-conditioning, but even with air-conditioning, there is still a notable peak throughout the day, suggesting the current unit is struggling to maintain temperatures, even in the smallest scanning room (Figure 6c,d and Table 1).

**Figure 8:**
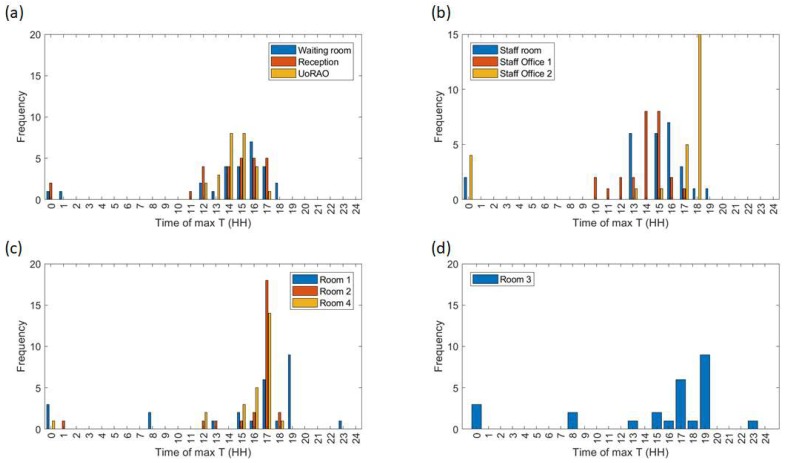
Hours in which the maximum daily temperatures recorded on the weekdays occur (Figure 9). For clarity, similar rooms are plotted on one subplot, (**a**) Waiting room, Reception and the observatory (UoRAO), (**b**) Staff room and Staff offices, (**c**) Room 1, 2, 4 and (**d**) Room 3. All plots have the same axis.

**Figure 9:**
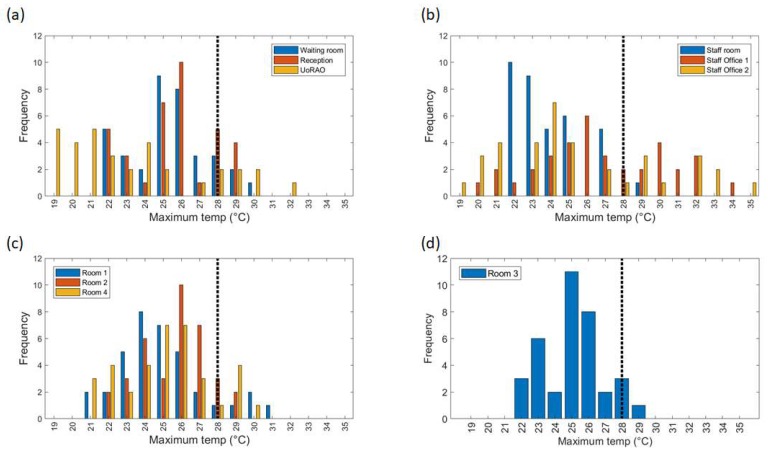
Maximum daily temperatures recorded in all rooms across all weekday measurements. For clarity, similar rooms are plotted on one subplot (**a**) Waiting room, Reception and the observatory (UoRAO), (**b**) Staff room and Staff offices, (**c**) Room 1, 2, 4 and (**d**) Room 3. All plots have the same axis. The vertical dashed line denotes an upper threshold of 28 °C.

Removing the weekend data and averaging over all weekday data further highlights the differing behaviour of the scanning rooms throughout clinic times (Figure 7c,d). On average staff office 1 experiences a peak in mean temperatures at around 15:00, whereas Staff office 2 peaks between 18:00 and 19:00 (Figure 7b, Figure 8b), with some of the highest maximum temperatures (30–35 °C) being recorded here (Figure 9b). This is most likely due to the solar gain of those offices. Based on room positioning time of year, Staff office 1 would have maximum solar gain between 11:00–14:00 (south-facing) and Staff office 2 (west-facing) 16:00–19:00. Staff office 2 also experiences the lowest temperatures, which is a concern for the winter months, as, despite windows being closed, it is a similar temperature to UoRAO temperatures during the evening (Figure 7b). More research into occupant adaptation and behaviour is required.

The staff room has a peak in temperature at around 14:00, linked to anthropogenically created heat from lunch breaks between 12:00 and 14:00 (Figure 7b, Figure 8b). The waiting room and reception areas experience a slow rise in temperature throughout clinic times (Figure 7a), with maximum temperatures occurring most frequently between 12:00 and 17:00, in part due to the presence of people (Figure 8a). This rise in temperature is less steep than the scan rooms (Figure 7c) due to the proximity to the entrance doors, less equipment, and the influence of the portable air-conditioning unit installed within the waiting area.

A large variation is seen in the daily average temperatures for scanning rooms 1, 2, and 4, with all tending towards a peak in mean temperatures at 17:00 (Figure 8c), an accumulation of high external temperatures, waste heat from equipment and anthropogenic heat (Figure 7c). Scan room 3’s mean temperature is kept fairly constant due to the air-conditioning unit (operated by staff, with heat extracted to the roof) with its most frequent daily maximum temperatures being 1–2 °C lower than the other rooms (Figure 9c,d). Differences between rooms 1, 2, and 4 are likely due to differing usage patterns, size, staff preference, location of different equipment, and differing structure (Figure 2). The time of the daily maximum temperatures of Scan room 3 is slightly later than the other rooms, due to the air-conditioning unit being turned off after clinic hours and the room returning to equilibrium (Figure 8d).

Maximum temperatures occurring around 23:00 to 01:00 h were not due to the heating system, updating equipment or out-of-hours use of the area. These occurrences mostly occur on a day where conditions changed from sunny to overcast (e.g., 08/08/2019 after the period with the highest maximum temperatures, Figure 6 and the peak external temperature dropped significantly, with the late-night temperature being caused by heat storage, especially if rooms are closed off with little ventilation.

Focusing just on the scan rooms, the clinics run five days a week from 09:00–17:00 each day and accounting for bank holidays, this equates to ~20 h out of 2016 clinic hours above 28 °C per year. Two-hundred-and-twenty-five clinic hours were measured over the course of this study. The results of the Staff room (S) and Scan room 1 (SR1) suggest that high temperatures within those rooms are not persistent and may be caused by equipment being appropriately powered down when not in use. The clinic hours above 28 °C in the observation period are divided by the number of clinic hours per year to highlight the impact of this single high-temperature period on the overall yearly data. All rooms aside Scan room 1 (SR1), the Staff room, and Scan room 3 (SR3) breach the threshold set by CIBSE for a year within this study period, which does not include other heatwave conditions declared earlier and later in the year (Table 2, [38]). The increased percentage of the reception compared to the waiting room is likely due to waste heat from PCs and increased distance from the entrance doors.

## 4. Discussion

This study has focused on the Maternity and Gynaecology (M and G) building’s ultrasound scanning rooms due to patient type acute sensitivity and greatest concern from staff working in the area. The causes of the M and G building’s poor performance are numerous, and whilst some compromises and improvements can be made there is no solution that does not require significant investment. The building is constructed primarily from concrete and asbestos, both of which have high thermal mass. When coupled with high solar gain through absence of façade shading and poor ventilation in clinical rooms, indoor temperatures exceeding recommended levels can be expected to be an increasing occurrence [36] (Figure 9). However, the results of overheating are not unique to RBH. For example, Ref. [42] identified overheating (particularly night-overheating) as an issue in a mechanically ventilated 1970s maternity unit, which with climate projections was estimated to always breach CIBSE overheating limits within certain rooms without intervention. Patients have also complained about overheating of maternity facilities in literature as early as the 1980s [43]. This is the first study looking at the Maternity and Gynaecology (M and G) building where the pregnant women may be most vulnerable population in hospital to heatwaves.

Solutions to overheating in hospitals are often focused across the overall hospital site, which whilst overheating needs to be addressed in all buildings, building-specific solutions may be quicker to implement and reduce indoor temperatures in the short-term, called low-hanging fruits. Ref. [42] suggests a range of solutions for the building as a result of the study, highlighting the importance of understanding how the building performs and working within limitations. The overall layout of the building is critical to ensure adequate ventilation.

All solutions were required to align with the core values of the RBH: a dedication to the continuous improvement of people and services and a commitment to future-proofing and putting quality patient care at the forefront. However, they are also likely to be applicable to other NHS trusts and have been reported in a generalized way.

The hierarchy of intervention effectiveness suggests that technological, system-focused interventions (e.g., mechanical ventilation) are more effective than those relying on changes in human behaviour [44]. However, at a systems level, several limiting factors influence the implication of solutions, these include but are not limited to budget, patient safety and privacy, complexity, maintenance, hospital reputation, and building limitations. The changing climate must also be considered with temperatures in the South-East predicted to rise by 2–4 °C by 2039 and 3–5 °C by 2059 [1]. Ref. [1] also predicts that summers as hot as 2018 will increase in probability from <10% to an estimated 10–25%, meaning that overheating will become more frequent.

Whilst some short-term solutions are suggested within the NHS Royal Berkshire Foundation Hospital Trust’s Adverse weather guidelines, these are only triggered once external temperature requirements (31 °C daytime temperature and 16 °C max night time temperature [18]) are met, whereas the indoor conditions become a risk before these conditions are met. As such, preparation within this area should begin at the start of the summer period and remain in place until October for staff and patient safety. Similar pro-active, rather than reactive behaviour may also be of benefit for other buildings and other Trusts [17].

Often the central scanning rooms are closed off for patient privacy, further reducing ventilation from doors and nearby windows. The anthropogenic waste heat, odour and CO_2_ further exacerbate the problem. For rooms where air-conditioning is in place, doors should remain closed to ensure that cooling is effective. Waste heat from old bulbs may also contribute, with changing to LED (light-emitting diode) bulbs a potential solution. Again, these are broadly applicable to similar working environments.

One of the key problems at RBH identified by staff was the centralised control and slow response of the ventilation systems. Within the M&G building, there are multiple clinics, wards and services which require different climates. For example, wards are often kept warmer for elderly patients and premature babies, whereas this may be too warm for clinic patients. Diligent maintenance of existing HVAC systems can also reduce energy wastage, operating costs, and CO_2_ emissions, a solution not just limited to the RBH.

Another highlighted problem is the lack of data and evidence about room temperatures, which prompted the department to buy thermometers for certain rooms. Whilst these instruments may be cheap and inaccurate, if installed correctly they provide staff with a guideline of when to stop using temperature-sensitive disinfectants and when to pre-emptively trigger local heatwave measures. Having estates departments deploy measurements in at-risk areas will allow for a greater understanding of the unique problems faced in each hospital area.

### 4.1. Ventilation and Cooling

Natural ventilation is not a viable solution in this area of the RBH, due to the central position of the at-risk scanning rooms within the building (Figure 2). Also, within heatwave conditions, it is expected that air-pollution also increases, adversely affecting those with respiratory conditions [18]. Refs. [24,45,46] highlight that there are few examples of innovative natural ventilation/passive cooling strategies being used in hospital buildings, due to risk-adverse procurement and tight budget constraints. Noise may also prevent windows and doors from being opened. Whilst this study has focused on temperature, other factors, such as airflow, moisture, and odour also influence thermal comfort and may also need to be considered for specific patient groups. Other more complex ventilation solutions are described in [45].

Leaving internal doors open where possible overnight will allow for cooling across the entire building, though security concerns may prevent this. Another option, dependent on clinic demand, is to allow for the rotation of rooms used for scanning throughout the day, to equalise the exposure amongst staff and to ensure that they have some relief from the heat. Rooms not in use can have equipment switched off to prevent overheating. This solution could be applied NHS wide, as long as the unique heating patterns of buildings are understood.

The use of desk, standing and ceiling fans may reduce reports of air staleness, a low-energy, low-cost and low intervention approach, these are dependent on health and safety, infection control and also the weight-bearing ability of the ceilings [25]. Temporary cooling is provided by portable air conditioning units as per the local heatwave plan, but these require a location and maintenance plan to reduce infection risk and ensure waste heat is vented appropriately. Local staff are not instructed on how to effectively use the units, often rendering them ineffective over time. Portable air-conditioning units have a limited range, and should be positioned in areas of (a) most need/highest temperatures (e.g., Scan room 3) or (b) where most people will benefit from them (e.g., the waiting area).

A more permanent RBH specific solution would be to build air conditioning units into each scanning room, as they are effective at maintaining constant cooler temperatures. With traditional air-conditioning units, a maintenance plan, and a suitable design are essential to ensure that the units themselves are not an infection source due to mould build-up [47,48] and that waste heat is suitably vented away. Good practice in the maintenance and repair of existing energy services is relatively low cost and has an important role to play in improving the resilience of buildings [25]. Depending on the budget chilled beams are also a potential option [49].

Safe storage of Tristel wipes away from heat sources should also be encouraged and enforced, as these are often stored for convenience on the machines, which give out waste heat, leading to fumes being released. Providing specific storage solutions may aid prevent this.

### 4.2. People

Increasing patient’s awareness of poor conditions within the building if done correctly would not impact on the public opinion on the department. Repeat visitors are likely to take preparatory action and adjust their behaviours based on their previous experiences, but for new patients, the uncomfortable conditions may impact on their perception of the service provided [43]. Including a warning of the high temperatures in clinic letters may help. Another option may be to provide more general heatwave coping tips and to remind patients to dress appropriately for the weather, wear easily removable layers and to bring fluids. Again, this could be standardised for the entire hospital and utilised NHS wide.

When the patients are in the building, easy access to drinking-safe water would aid in ensuring that patient’s stay hydrated. In this test study, whilst there are shops nearby (Figure 2), it is not possible for patients to visit whilst remaining in sight or hearing distance of the waiting area, and it is not clear whether taps in toilets are safe to drink. Lidded jugs of water or a water machine (pre-existing) are possibilities. Disposable cups and other consumables should be closely monitored in the risk period, in order to ensure that patients can actually access the water. This should be given as a dedicated role to a member of staff. An option which does not require extra resource is the installation of a piped water-fountain. This is especially important if the waiting time has increased, the waiting area is busy or if the patient is attending a clinic in the afternoon, as these were often when the highest internal temperatures occurred (Figure 7 and Figure 8). This measure may also encourage staff to stay hydrated as they benefit from easy access to drinking water. Again, increasing water availability without increasing infection risk hospital-wide may be of benefit from a health perspective, especially if nationwide guidelines are created to facilitate easy installation.

Identifying the most vulnerable patients and ensuring that their appointments are in the morning may also reduce heat-risk exposure to those individuals both during their stay and on their journey to and from the Royal Berkshire Hospital. Another option is to ensure that they are seen in the lower-temperature rooms if possible. Signs instructing patients to inform a member of staff if they feel unwell may encourage patients to seek pre-emptive help. Instructions for staff as to how to rapidly cool patients and how to manage their own heat exposure should also be provided. Again, this could be applicable to all hospitals, though the timings of cooler appointments will differ depending on building and location.

Setting up a cool room (<26 °C) [18], in line with the actions outlined in the NHS Royal Berkshire Hospital Foundation Trust’s Adverse weather guidelines at all times over the summer period will aid in ensuring patient and staff risk is reduced (Level 1 heatwave action in [18]).

Uniforms pose a particular challenge in successfully balancing the needs of adequate infection control, personal protection and protecting the individual from excess heat gain. Experience from departments such as Special Care Baby Units where high temperatures are the norm may be useful [26].

## 5. Conclusions

The Maternity and Gynaecology building at the Royal Berkshire Hospital suffers from overheating issues throughout the year, not just during the summer, with overheating events likely to increase in length and frequency due to climate change [1]. Waiting areas, receptions, and scan rooms all breached overheating guidelines on indoor temperature limits within the 35-day measurement period. Maximum temperatures occurred during the afternoon, though the timing of these vary depending on room location, with those exposed experiencing larger peaks related to solar gain at differing times compared to internal rooms, where temperatures rose quicker due to poor ventilation. The effect of anthropogenically generated heat is highly significant and should be considered when optimising patient flow. The indoor environment and the external environment are not well coupled, especially for the more central rooms with high indoor heat generation, meaning heatwave conditions based on external temperatures may not always be relevant, with preventative measures being triggered too late.

The overheating challenges facing the NHS have no single cookie-cutter solution due to the wide variety of building stock. This paper has focused on an area of a single building within one hospital and has covered solutions from a low hanging fruit level (such as clothing advice in clinic letters and improved patient access to water) to a hospital site level (improved control of ventilation by estates services) with the aim of aiding organisations facing similar over-heating problems. A core issue is a lack of localised information/data for hospitals as knowing when the overheating will occur will enable precautionary measures, such as scheduling the most vulnerable patients (e.g., those who are most advanced in their pregnancy) at typically cooler times in the morning. However, this solution suffers from system-wide problems due to patient data confidentiality issues. Some simple solutions are summarised here:

- Adjustment of clinic times to cooler periods (in this case the morning) for especially vulnerable patients.
- Improved signage and correspondence with patients to encourage dressing suitably (easily removable layers) for high temperatures.
- Cycling of used rooms to allow rooms to cool and to ensure that staff have some guaranteed relief from poor conditions.
- Procurement of fans to improve air-movement and if possible, cooling equipment.
- A thorough maintenance schedule that covers any temporary HVAC equipment brought in during heatwaves.
- Ensure the easy availability of water to staff and patients, with a dedicated staff member ensuring it is accessible.
- Set up a cool-room (<26 °C) to provide relief from the heat at all times, not just during heatwave conditions.

Since this case is only one in an array of similar cases, it is clear from the short measurement period that if the RBH Maternity and Gynaecology building is breaching overheating thresholds in 2018 then even by 2030 this will be endemic on a national level. Whilst air-conditioning units help to lower the indoor temperatures, this is in direct contradiction to NHS sustainable initiatives. Localised, low carbon solutions may aid in reducing the impact of a changing climate. Future work should consider the full exposure of individual patients as they make their way to and around the hospital as differing routes will lead to different exposures. Local air quality, CO_2_ concentrations and airflow should all be considered in conjunction with temperature for a range of hospital buildings and patient types.

## Figures and Tables

**Table 1:** Symbols used for sensors, room size and location within the test area. Locations are shown in Figure 2.

Sensor	Room	Room Size (m^2^)	Sensor	Room	Room Size (m^2^)
SO1	Staff Office 1	18.66	SR1	Scan Room 1	13.96
SO2	Staff Office 2	8.70	SR2	Scan Room 2	18.50
R	Reception	8.95	SR3	Scan Room 3	11.88
W	Waiting room	35.77	SR4	Scan Room 4	12.39
S	Staff Room	17.06			

**Table 2:** The number of clinic hours breaching an average temperature of 28 °C during the observed and for as a percentage of the total yearly clinic time.

Room (Label on Figure 2)	Hours above 28 °C	Percentage of Clinic Time above 28 °C in Observation Period	Percentage of Yearly Clinic Time above 28 °C
Waiting room (W)	36	16%	1.79%
Reception (R)	44	20%	2.18%
Staff room (S)	2	0.9%	0.1%
Staff Office 1 (SO1)	50	22%	2.5%
Staff Office 2 (SO2)	23	10%	1.14%
Scan Room 1 (SR1)	2	0.9%	0.1%
Scan Room 2 (SR2)	31	14%	1.54%
Scan Room 4 (SR3)	34	15%	1.69%
Scan Room 3 (SR4)	16	7%	0.79%
